# Impact of physiological parameters on the parotid gland fat fraction in a normal population

**DOI:** 10.1038/s41598-023-28193-z

**Published:** 2023-01-18

**Authors:** Ari Lee, Yoon Joo Choi, Kug Jin Jeon, Sang-Sun Han, Chena Lee

**Affiliations:** Department of Oral and Maxillofacial Radiology, Yonsei University College of Dentistry, 50-1 Yonsei-ro Seodaemun-gu, Seoul, 03722 Republic of Korea

**Keywords:** Anatomy, Magnetic resonance imaging

## Abstract

Quantifying physiological fat tissue in the organs is important to further assess the organ’s pathologic status. This study aimed to investigate the impact of body mass index (BMI), age, and sex on the fat fraction of normal parotid glands. Patients undergoing magnetic resonance imaging (MRI) of iterative decomposition of water and fat with echo asymmetry and least squares estimation (IDEAL-IQ) due to non-salivary gland-related disease were reviewed. Clinical information of individual patients was categorized into groups based on BMI (under/normal/overweight), age (age I/age II/age III), and sex (female/male) and an inter-group comparison of the fat fraction values of both parotid glands was conducted. Overall, in the 626 parotid glands analyzed, the fat fraction of the gland was 35.80%. The mean fat fraction value increased with BMI (30.23%, 35.74%, and 46.61% in the underweight, normal and overweight groups, respectively [p < 0.01]) and age (32.42%, 36.20%, and 41.94% in the age I, II, and III groups, respectively [p < 0.01]). The fat content of normal parotid glands varies significantly depending on the body mass and age regardless of sex. Therefore, the patient’s age and body mass should be considered when evaluating fatty change in the parotid glands in imaging results.

## Introduction

The parenchyma of the salivary gland is composed of secretory and connective tissues^1^. As gland dysfunction progresses, histological components change. Prominent changes can be observed when the increased proportion of adipose tissue displaces the acinar cells, the secretory part of the gland^1,2^. Thus, the adipose tissue contained in the glands is considered an important indicator of gland dysfunction^3–6^. As the fat fraction is considered an indicator of gland function, different measurement methods using various imaging techniques have been introduced^7–9^.

In addition, the internal body organs are continually changing their tissue composition according to physiological conditions^10^. Thus, many researchers have studied the fat fraction alteration of organs, such as the liver or bone marrow, according to physiological factors including age, sex, or body mass^11–13^. However, few studies have focused on the fat fractional changes in the salivary glands related to physiological factors. Studies on the salivary gland are not exhaustive, and they have revealed a weak to moderate linear correlation between physiological factors and the fat fraction until recently^14,15^. Quantification of alteration in the fat fraction of the salivary gland from baseline is needed prior to studying gland dysfunction.

Fat quantification can be performed based on various techniques of magnetic resonance imaging (MRI). Among them, Iterative Decomposition of water and fat with Echo Asymmetry and Least-squares estimation (IDEAL-IQ) is known as a reliable and precise method for fat quantification^16^. Basically, this method uses magnetic resonance spectroscopy and measures the proportion of metabolites, including lipid and water, according to their different resonance frequency in the magnetic field. The iterative least-squares decomposition algorithm calculates the fat and water value of the individual pixels in the entire image and produces a fat fraction map image^17^. The IDEAL-IQ is a recently developed method that separates the water-fat signal and simultaneously estimates T2* based on a multiecho chemical shift. The technique is known to calculate fat, which has multiple spectral peaks, more accurately^18^. In addition, a previous study reported the successful measurement of fat saturation in the head and neck with metal artifacts having little effect on the MR image^14^. Currently, this method is widely applied clinically and in the research field to analyze the liver or bone marrow of the proximal femur^13,19,20^. However, few researchers have used this method to study the salivary gland fat fraction^14,15^.

It is necessary to explore the fat fractional change in normal parotid glands according to physiological parameters in a large population. Therefore, this study aimed to investigate the impact of body mass, age, and sex on the fat fraction of the normally functioning parotid gland. In addition, the difference in the fat content of the gland according to individual physiological factors was suggested using the IDEAL-IQ technique.

## Methods

This study was approved by the institutional review board (IRB) of Yonsei University Dental Hospital (No. 2-2021-0058) and was conducted in accordance with the relevant guidelines and ethical regulations. Due to the retrospective aspect of this study, the need for informed consent was waived by the by the IRB of Yonsei University Dental Hospital.

### Study population

**Figure 1:**
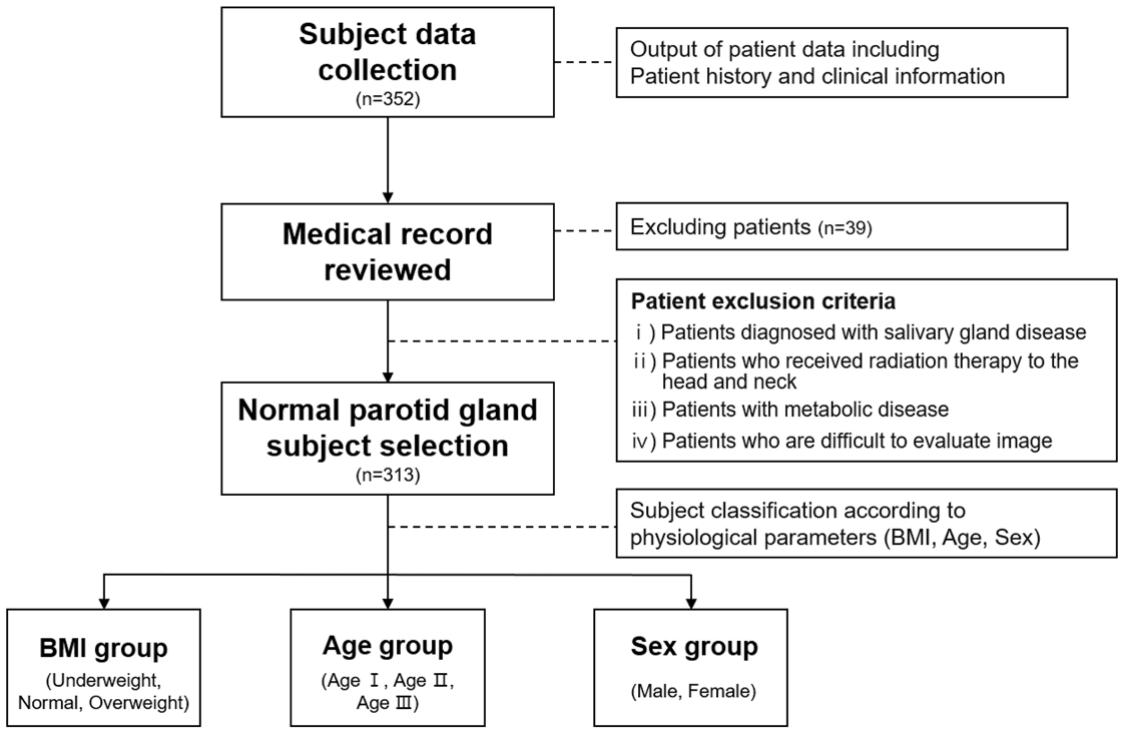
Patient inclusion and exclusion chart.

Patients who underwent MRI examination from July to December 2020 at our institution were included in the study. A thorough chart review was performed to collect general clinical information; details on age, sex, body weight, and height; and current or previous history of any compromised disease. The exclusion criteria were as follows: patients (i) diagnosed with salivary gland tumors, sialadenitis, or auto-immune diseases, (ii) who underwent radiation therapy due to head and neck cancer or symptoms of oral dryness, (iii) without metabolic diseases such as hypertension and diabetes, and (iv) for whom it was difficult to evaluate and measure owing to degraded image quality caused by artifact. The IDEAL-IQ images covering the complete volume of the parotid gland without any imaging errors were included in this study. The process of selecting the patients has been explained in detail in a flow chart (Fig. 1).

### Scan parameters

All MRI scans were acquired using a 3.0 T scanner (Pioneer; GE Healthcare, Waukesha, WI, USA) with a 16-channel flex large coil. IDEAL-IQ and T2-weighted images in the axial view were included in this study. The imaging parameters of IDEAL-IQ were as follows: echo time, 1.00, 2.04, 3.08, 4.12, 5.16, 6.20 ms; echo train length, 3; repetition time, 10.52 to 10.63 ms; bandwidth, 868.047 kHz; NEX, 1.0; field of view, 240 × 240 mm; slice thickness, 4.0 mm; scan time, 1 min. flip angle, 4°; and matrix, 160 × 160. The imaging parameters of T2-weighted image were as follows: echo time, 85 ms; echo train length, 9; repetition time, 3100 ms; bandwidth, 83.33 kHz; NEX, 1.0; field of view, 230 × 230 mm; slice thickness, 4.0 mm; scan time, 2:17 min; flip angle, 111; and matrix, 380 × 320.

### Physiological parameters

Body mass index (BMI), age, and sex were considered as the physiological parameters.

#### Grouping according to body mass index

BMI was calculated using body weight and height (BMI = weight [kg]/height^2^ [m^2^]) and was categorized into three groups based on the World Health Organization report. BMI < 18.50 was defined as underweight, 18.50–24.99 as normal weight, and > 25.00 as overweight^21^.

#### Grouping according to age

Three age groups were also established as follows: age I, 10–29 years; age II, 30–49 years; and age III, > 50 years.

#### Grouping according to sex

Two groups were defined according to biological sex of male and female.

### Image analysis

**Figure 2:**
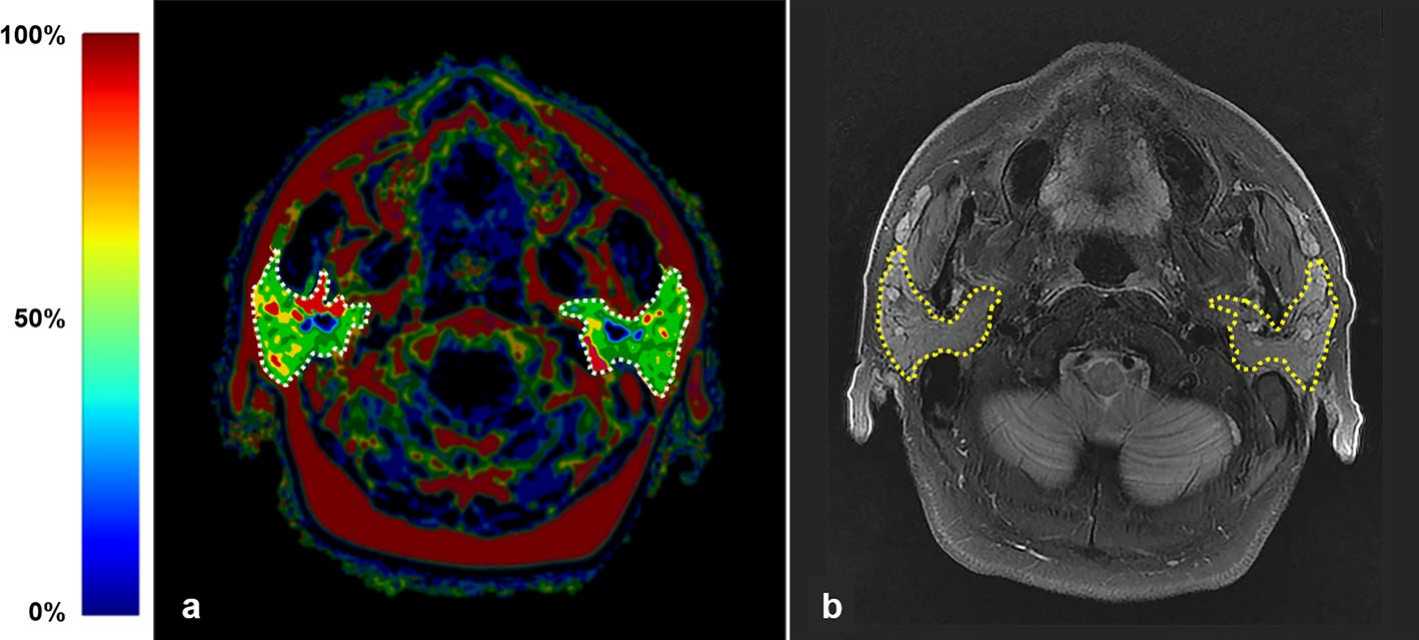
Magnetic resonance imaging presenting the largest axial area of both parotid glands. (**a**) The fat fraction map image with the selected regions of interest (white dotted line) on the parotid gland. (**b**) T2-weighted image with fat saturation. Note that the vessels and duct are excluded and only the parenchyma is included. The selected regions of interest are indicated by yellow dotted lines.

The IDEAL-IQ was analyzed by two observers specialized in oral and maxillofacial radiology (one specialist and one graduate student) (Fig. 2a). Before establishing the study design, all parotid gland image slices were measured in 30 patients to confirm the concordance of the fat fraction according to gland location. The axial image slice showing the largest gland size was selected. The measurement section was determined by two observers through discussion. The observers then determined and selected the region-of-interest (ROI) by analyzing pilot cases. The ROIs were manually drawn along the border of the gland using the PACS viewer free-hand drawing tool (ZeTTA PACS, TaeYoung Soft, Anyang, Korea, http://taeyoungsof.com/ product01.php). Based on the T2-weighted images, non-parenchymal areas such as blood vessels were avoided (Fig. 2b); thereafter, the fat fraction was extracted within the determined ROI (Fig. 2a). Two observers performed the same measurement, and the first observer conducted the measurement twice after a 1-month interval for inter- and intra-observer reliability analysis.

### Statistical analyses

The physiological parameters and fat fraction were examined for normality using the Kolmogorov–Smirnov test. The continuous variables are presented as the mean and standard deviation (SD). The correlation between BMI or age and fat fraction was confirmed through Pearson's correlation analysis. Analysis of variance was used to compare the fat fraction with BMI or age group. For post-hoc analysis, the Bonferroni method was used. An independent t-test was used to analyze the male and female sex groups. To confirm that there was no difference between the left and right sides, a paired-t test was performed. The criterion for statistical significance was set at p < 0.05. The intraclass correlation coefficient (ICC) with 95% confidence intervals (CIs) were evaluated for inter- and intra-observer concordance. A correlation coefficient (r) of < 0.2 was regarded as weak reliability, 0.3–0.5 as moderate reliability, 0.6–0.8 as strong reliability, and 1.0 as perfect reliability^22^. The Bland–Altman plot was used to ascertain the distribution of intra- and inter-observer agreement. Statistical analyses of the data were performed using the Statistical Package for the Social Sciences version 26.0 for Windows (IBM Corporation. Armonk, NY, USA).

## Results

**Figure 3:**
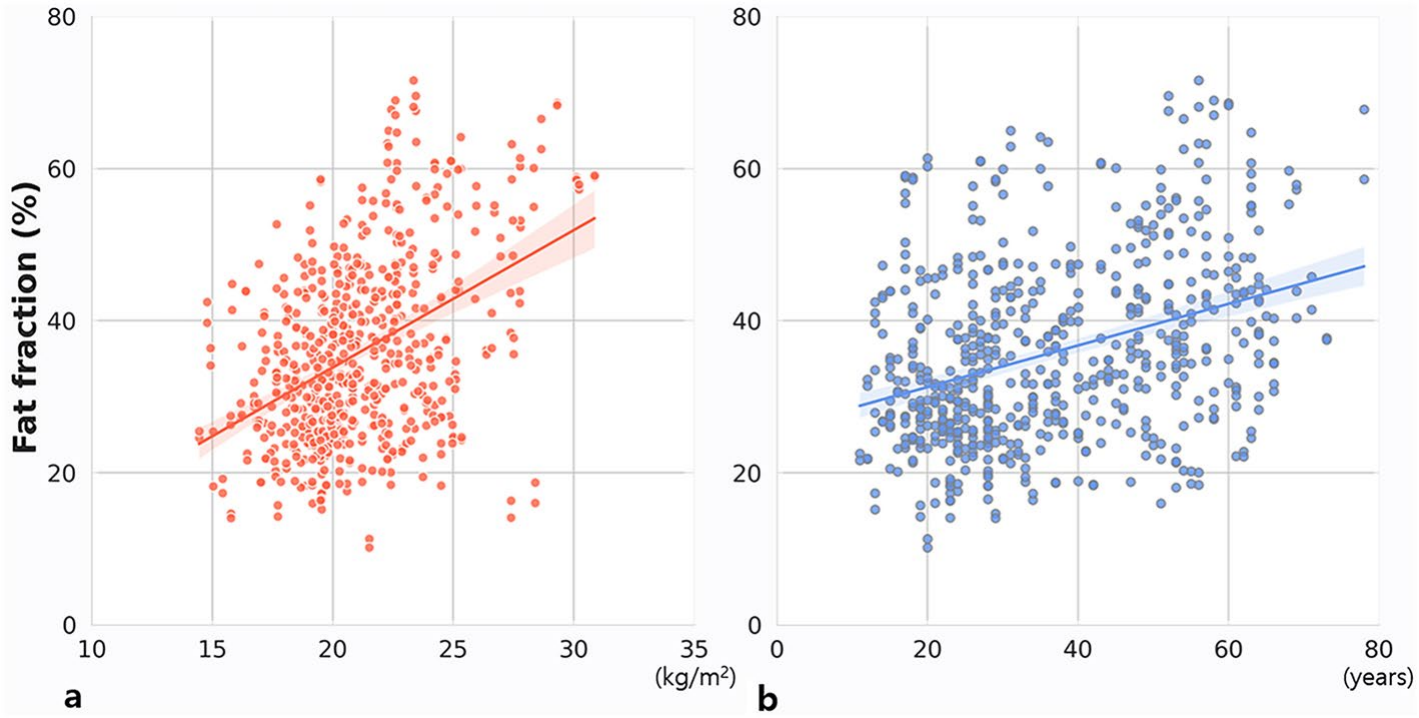
Scatter plot of fat fraction values with the (**a**) body mass index and (**b**) age.

**Figure 4:**
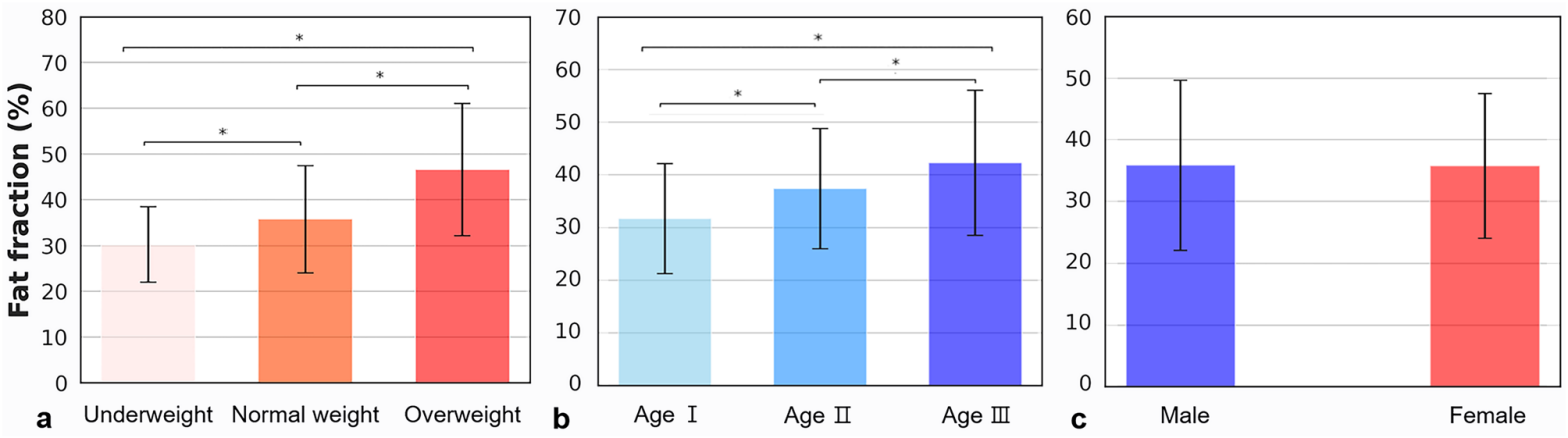
Bar plot of the fat fraction associated with the physiological parameters. (**a**) The body mass index (BMI) groups show statistically different fat fraction among all groups (underweight, BMI < 18.50; normal weight, 18.50–24.99; and overweight, BMI > 25.00). (**b**) The age groups show significant difference in the fat fraction among all groups (age I, 10–29 years; age II, 30–49 years; and age III, > 50 years). (**c**) The sex groups show no difference in the fat fraction (*p < 0.01).

**Figure 5:**
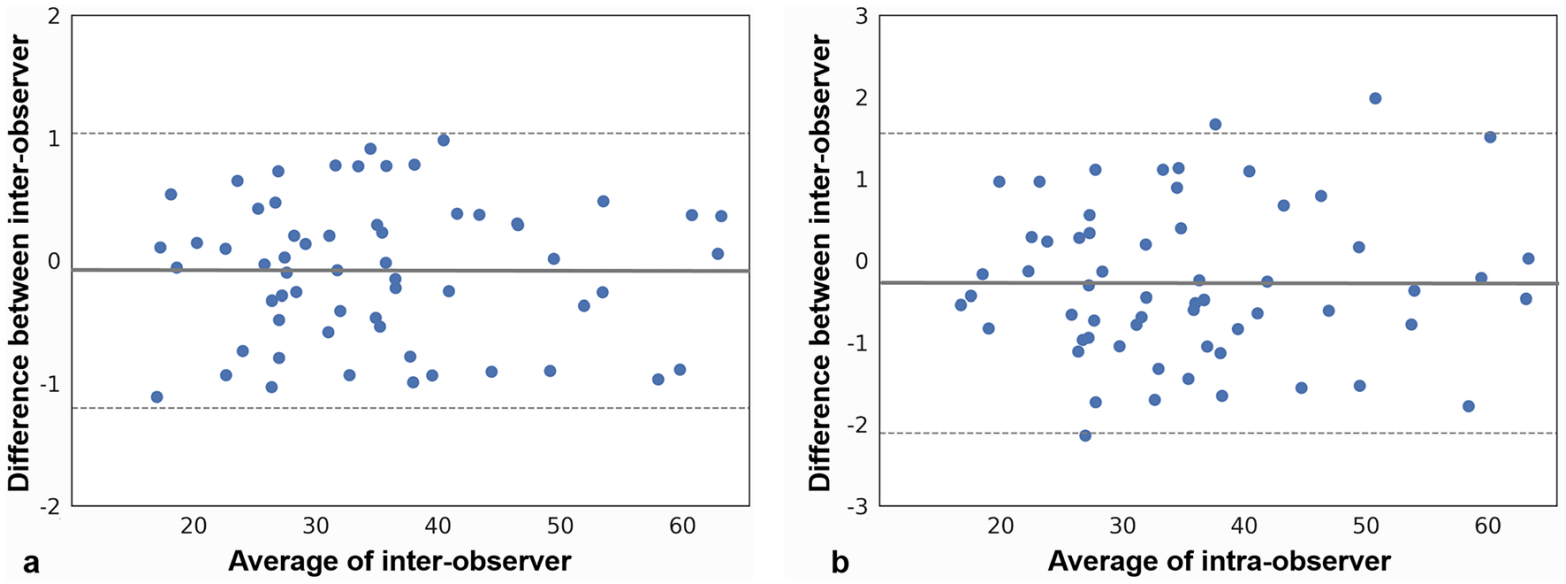
Bland–Altman plots evaluating inter-observer and intra-observer agreement for the fat fraction measurement using magnetic resonance imaging, (**a**) inter-observer agreement (**b**) intra-observer agreement. Dotted line, 95% confidence interval; solid line, mean of the differences (= bias) between the two observers.

A total of 626 glands (313 patients) were included in the study. The physiologic parameters of the patients were age (36.69 ± 15.70 years), BMI (21.22 ± 2.98 kg/m^2^), and sex (114 men and 512 women). Both BMI and age showed positive moderate correlation with the parotid gland fat fraction (Fig. 3; BMI, r = 0.433; age, r = 0.356).The fat fraction increased in the following order: underweight (30.23%), normal weight (35.74%), and overweight (46.61%). The fat fraction was the highest in the age III (42.33%), followed by age II (37.42%), and age I (31.70%) group patients. The difference between groups for both factors was statistically significant (p < 0.05; Table 1, Fig. 4). The fat fraction in men (35.88%) and women (35.78%) showed no statistical difference (p = 0.944; Table 1). The fat fraction of the total parotid glands was 35.80 ± 12.11% (right, 35.97 ± 12.02%; left, 35.62 ± 12.23%), with no significant difference between the right and left side (p = 0.102). The intra- and inter-observer ICCs for measurement were excellent at 0.998 (95% CI 0.996–0.998) and 0.954 (95% CI 0.943–0.963), respectively. The Bland–Altman plot of inter-observer showed agreement within the 95% confidence limit (Fig. 5a), and the plot of intra- observer mostly presented agreement with a few errors between the observers (Fig. 5b).

**Table 1:** Comparison of fat fraction according to body mass index, age, and sex.

Physiological parameter		Number of samples	Mean ± standard deviation (%)	p-value
BMI	Underweight	108	30.23 ± 8.24	0.000*
	Normal weight	460	35.74 ± 11.67	
	Overweight	58	46.61 ± 14.42	
AGE	Age I	274	31.70 ± 10.43	0.000*
	Age II	192	37.42 ± 11.42	
	Age III	160	42.33 ± 13.73	
SEX	Male	114	35.88 ± 13.78	0.944

Statistically significant difference at *p < 0.05.

## Discussion

The influence of physiological parameters on the fat fraction of body organs such as the liver or bone marrow has been reported earlier^11–13^. Consistent with the previous findings, the current study elucidated that the parotid gland was affected by body mass and age. However, sex difference did not contribute significantly to the fat content of the gland parenchyma. These findings support that the fat fraction of the parotid gland may show critical level of change, regardless of dysfunction.

In terms of persistence of gland dysfunction, the acinus tissue undergoes atrophic changes, and is replaced by the adipose tissue^3–5^. Thus, an increase in the fat proportion within the gland is generally considered a pathological change^3^. However, this study confirmed that even in normal parotid glands, the fat proportion increased according to body mass and age. Therefore, we recommend that body mass and age be considered when diagnosing salivary gland dysfunction based on fat fraction measurement.

In this study, among the physiological factors, body mass substantially influenced the level of fat in the gland. A previous study by Su et al. reported that BMI (r = 0.431–0.433) showed a stronger correlation with parotid fat amount than did age (r = 0.369–0.432)^15^. Additionally, Chang et al. used linear regression analysis to show that parotid gland fat content positively correlated with age and BMI (p = 0.002)^23^. The current study results were consistent with Chang et al.’s results. In addition, in studies concerning other body organs, including the proximal femur bone marrow and liver, BMI showed a correlation with fat in the individual organs^11–13^. Moreover, the correlation of BMI with fat in the body organs was stronger in the salivary gland than in the bone marrow and liver^11,13,15^. The positive correlation was stronger when the water content of parotid gland was measured according to age and BMI^23^. The apparent diffusion coefficient mapping is a feasible sequence to measure the water content as a functional evaluation of the gland. However, the limitation of this imaging technique is the difficulty in specifying the precise parotid gland region owing to low image resolution.

Age was also found to be a factor significantly influencing the fat content of the parotid gland in the present study. The fat fraction of the gland increased with age. In addition, different age groups showed significantly different fat content. The fat fraction of the salivary gland has long been investigated with respect to aging, but with controversial results^24,25^. From the 1980s, Scott and Atkinson et al. suggested salivary gland hypofunction with aging and that glands undergo fatty degeneration^26,27^, whereas more recent studies have suggested that the fat fraction of the gland increases with aging, even without hypofunction^14,15^. Our study results support the recent findings that the gland fat fraction differs between age groups even in normally functioning glands.

The controversial results regarding aging and fatty change of the gland can be inferred from several previous studies^2,15,19^. Su et al. and Chang et al. reported a linearly increasing parotid gland fat fraction with age; however, the distribution showed a significantly wide range for each age^14,15^. Another study that measured the T1 and T2 relaxation time of the gland according to age also mentioned that the parotid gland exhibited large inter-subject variability^28^. One of the reasons for the controversial result is the difficulty in defining gland hypofunction, especially in older age. To study more accurate fat measurement of the gland, completely controlled normal functioning gland should be defined primarily in further research.

Nevertheless, the difference between sexes was insignificant in the evaluation of the fat fraction in one of the previous studies and the current study^14,15^. This is inconsistent with another study that showed a significant difference in the water content of parotid gland between men and women^23^. This discrepancy in results is probably due to the differences in the sample size, age, and sex distribution. The previous study used a smaller sample size and the age distribution was higher than in our study. Moreover, we used an imbalanced male to female ratio, which may have also contributed to the difference in study results.

Compared to the previous studies using the same imaging method, the current study had a large sample size^14,15^. Chang et al.^14^ and Su et al.^15^ performed the study with 114 and 87 study subjects, respectively. The current study was conducted with a study subject number approximately three-times larger; generally, errors due to heterogeneity between individuals can be reduced by adopting larger sample sizes. Considering that parotid gland shows large variation in size in different individuals, a large sample size was thought to contribute to more accurate study results^29,30^.

There were a few limitations to the current study. For example, complete screening of patients with mild gland hypofunction was not feasible. Because of the retrospective nature of the study, salivation tests were not performed and patients unaware of their gland hypofunction may have been included in the study. Therefore, further studies with strictly controlled study conditions are needed to validate our results. In addition, only the representative section was measured to efficiently confirm the fat fraction of the parotid gland in a large sample size. Since all the study subjects were normal and healthy, we assumed that a section may represent overall gland volume; however, it is possible that the fat fraction may be more accurate in detail when the volume of the gland is measured.

Another limitation of the current study was that among the major salivary glands, only the parotid gland was investigated. Most of the patients included in the current study were patients with temporomandibular joint disease, and the submandibular or sublingual glands were only partly shown in the images in most of the cases. It would be meaningful if the submandibular and the sublingual glands can also be included in further research to elucidate the relative gland function, fatty change, and physiological factors.

In addition, the current study emphasized the physiological parameters and their influence on the parenchyma of parotid gland using a robust method with a large sample size. As this study concludes that BMI and age have an impact on the parotid gland fat fraction, further study should be performed to find imaging evidence of parenchymal change, based on multi-variables including systemic disease, and medication.

## Conclusions

The fat content of normal parotid glands can vary significantly depending on age and body mass, and sex is not an influencing factor. Therefore, when evaluating the fatty change in the salivary glands in imaging results, the patient’s age and body mass should be considered. A careful approach is needed when evaluating the parotid gland based on the fat fractional changes in the images, especially in overweight or older patients.

## Data Availability

The datasets acquired during and/or analyzed during the current study are available from the corresponding author on reasonable request.
